# The prospects of community-based natural resource management in Ghana: A case study of Zukpiri community resource management area

**DOI:** 10.1016/j.heliyon.2021.e08187

**Published:** 2021-10-15

**Authors:** Issah Baddianaah, Louis Baaweh

**Affiliations:** a Department of Environment and Sustainability Sciences, Faculty of Natural Resources and Environment, University for Development Studies, P. O. Box TL 1882, Tamale, Ghana; b Department of Environment and Resource Studies, Faculty of Integrated Development Studies, SD-Dombo University of Business and Integrated Development Studies, P.O. Box W64/Wa, Ghana

**Keywords:** Local communities, Natural resource management, Sustainable livelihood, CREMA

## Abstract

The Community Resource Management Area (CREMA) model was adopted in Ghana in the 1990s to help conserve and increase the forest area of Ghana. Since its adoption, little is known about the prospects of the model in the scientific literature. To fill this gap, this study examined the management strategies, benefits and challenges of the Zukpiri CREMA in the Upper West Region. Mixed methods research involving a survey of 190 households, seven (7) focus group discussions and key informant interviews were employed to collect data in seven (7) CREMA communities. The study found that local communities employed several strategies including the formation of community resource management committees, enactment of bye-laws and fines regarding the management and extraction of the CREMA resources. The CREMA has positively impacted the livelihoods of the inhabitants through the harvesting of non-timber forest products (NTFPs) and support from Governmental and Non-Governmental Organisations. Nonetheless, the CREMA is not without challenges. These include land tenure and the CREMA resource use conflicts. This study, therefore, argued that besides creating an enabling ecologically balanced environment, inhabitants of the CREMA communities are reaping the benefits of the CREMA in many ways. Hence, the Forestry Commission of Ghana should focus on aligning relevant livelihood strategies in line with the CREMA approach to further deepen local communities’ commitment to the conservation drive.

## 1 Introduction

Worldwide, forest landscapes contain the most important natural resources which provide essential services and products for human sustenance (Husseini et al., 2016; FAO, 2016b; Jiao et al., 2019; Mavhura and Mushure, 2019; Islam et al., 2019; Gilli et al., 2020; Kamwi et al., 2020). Forest and wildlife resources are crucial in supporting, maintaining and replenishing global ecosystems (FAO, 2014; 2016b; Baskent, 2020). Despite the significant contribution of forests to human wellbeing, forest and wildlife resources have been subjected to a continuous cycle of depletion, particularly in the Global South. According to the Food and Agriculture Organisation [FAO] (2014), an average of 13 million hectares of the global forest cover is depleted annually, and Africa alone accounts for about 3.4 million hectares. Africa occupies the second highest position in the global ranking of deforestation, attributed to population increase and the associated increase in consumption of forest and wildlife resources (Husseini et al., 2016; Amoah and Korle, 2020). Besides, other human-induced factors such as the conversion of forest landscapes into farmlands and settlements are highlighted (Osei-Wusu et al., 2020).

In Ghana, over seventy percent of the population depends on forest and wildlife resources for their livelihoods (Amoah and Korle, 2020). Thus, the forest sector has persistently accounted for about 6 percent of Ghana's Gross Domestic Product (GDP) over the past decade (Osei-mainoo, 2012). However, overexploitation of these resources (both flora and fauna) is causing a significant level of depletion (Amoah and Korle, 2020). The country recorded a decline in forest cover of about 1.6 million hectares between 2000 and 2010 (Marfo, 2010; Osei-mainoo, 2012). Further, the country's forest degradation occurred predominantly within the closed forest landscape. FAO (2016a) reported a reduction in closed forest from 2,317166 ha in 2000 to 1,785,802 ha in 2010 with a corresponding forest degradation rate of 45,931 ha per annum. This raises questions about the effectiveness of policies and regulations on natural resource management and sustainability in the country.

Like many countries across the world, Ghana has a set of policies and regulations on ownership, access, consumption and management of natural resources. Foremost, the constitution of Ghana explicitly vests ownership and control of natural resources in the President in trust for the people of Ghana (Oduro et al., 2012). To prevent overexploitation and illegal harvesting of forest and wildlife resources, a number of policies and legislation have been formulated by the state to ensure sustainable utilization and conservation of these resources (Table 1).

**Table 1:** Policy guidelines on forest and wildlife resources in Ghana.

Forestry Commission Act 1960;
Forest Improvement Act 1960;
Concession Act of 1962;
Forest Ordinance for the protection of forest including reserves of 1972;
Trees and timber regulation of 1983;
Administration of land degree of 1984;
Forest products inspection Bureau Law of 1985;
Forest protection Law of 1986;
Control and prevention of Bush Fires Law of 1990;
Trees and Timber regulation of 1991;
Forest and Wildlife Policy of 2012;
National Environmental Policy of 2014;
National Climate Change Policy of 2014 etc.

Source: Oduro et al. (2012), O'Connor et al. (2021)

These policies and legislative frameworks are arguably state-centred and do not address the local people's needs in terms of sustainable management of forests and wildlife resources (Marfo, 2010; Baruah, 2015; Baker et al., 2018). Besides, the implementation of these policies has persistently encountered a number of challenges (O'Connor et al., 2021). As a result, various decentralised forest management methods have been suggested as alternatives to the state-centred and top-down approaches to mitigate the menace of deforestation and forest degradation (Baruah, 2015; Baruah et al., 2016; Baker et al., 2018).

The Community-based Natural Resource Management (CNRM) model is one of the decentralised natural resource management methods that give greater opportunity for local people to manage the natural resources under their lands (Marfo, 2010; Baland and Das, 2010; Baruah, 2015; Baker et al., 2018; Gilli et al., 2020; Owusu-Ansah, 2020). It is generally recognized that natural resource areas that are managed by or together with local communities have lower levels of degradation of forest and wildlife resources (Baruah, 2015; Husseini et al., 2016; Baker et al., 2018; Addison et al., 2019). In connection to this, the 1994 and 2012 forest and wildlife policy documents emphasize participatory forest and wildlife management (Oduro et al., 2012). This further triggers Ghana's government interest to embrace and roll out more collaborative resource management programmes and policies like the Voluntary Partnership Agreement (VPA), Reducing Emissions from Deforestation and Forest Degradation Plus (REDD+), Modified Taungya systems and the Community Resource Management Areas (CREMAs) (Foli et al., 2018). All these natural resource management approaches strongly highlight the involvement of local communities in natural resource management (Baruah, 2015; Baruah et al., 2016; Adams et al., 2017).

The Zukpiri CREMA–the study setting extends into the Black Volta River that forms an international boundary between Ghana and Burkina Faso–showcasing a unique characteristic of an integrated approach of conserving forest, wildlife and aquatic resources within a defined landscape. The CREMA, since its establishment on August 19, 2011, has received both national and international prominence. In 2015, the Zukpiri CREMA was the 2^nd^ runner up in the United Nations Development Programme (UNDP) Global Environmental Award in Washington D.C., USA. Additionally, in 2016, the United States Agency for International Development (USAID) evaluation of CREMAs in Northern Ghana rated Zukpiri CREMA first out of the 18 established CREMAs in Northern Ghana (Agyare, 2013). This suggests the need to explore the management strategies and livelihood prospects of the Zukpiri CREMA. CREMAs are sources of Non-Forest Timber Products (NTFPs) such as mushrooms, shea nuts, honey, medicinal products woodfuels including other edible fruits and wrapping leaves (Asare et al., 2013; Abukari and Mwalyosi, 2018; Baker et al., 2018; Foli et al., 2018; Murray and Agyare, 2018; Gilli et al., 2020), and also help in maintaining ecological balance (Foli et al., 2018; Murray and Agyare, 2018).

The significance of involving local communities in natural resource management cannot be downplayed. The CREMA approach is an essential participatory model capable of creating the right conditions for sustainably managing forest and wildlife resources by empowering local communities in forest and wildlife management. Relevant studies have argued that proper governance of natural resources must integrate and empower the local people to manage their natural resources (Baruah, 2015; Baruah et al., 2016). Thus, strong local leadership strategies and active involvement of the local people are needed to capture their views for better local level decision-making (Baker et al., 2018). While these are major highlights for effective natural resource management, natural resource management approaches at the local level are shaped by several factors. For instance, the governance and management of CREMAs in particular have a strong association with the cultural and religious practices of the local communities (Baker et al., 2018; Murray and Agyare, 2018).

Furthermore, community-based natural resource management paradigms come with a plethora of challenges–competing land uses, resource use conflicts, leadership conflicts, time and cost-intensive (Ostrom, 1992; Reed et al., 2016). And in spite of the fact that many communities are adopting the idea of co-managing their natural resources as a result of the implementation of the CREMA model in Ghana, key questions on how these CREMAs are managed, the challenges and contribution to livelihood and local community development remain pertinent. Thus, the overarching objective of this study was to explore the prospects of community-based natural resource management, using the Zukpiri CREMA as a case study. This study will contribute to the literature on scaling-up collaborative and participatory natural resource management strategies in developing countries (Baruah, 2015; Husseini et al., 2016; Adams et al., 2017; Baker et al., 2018; Duguma et al., 2018; Addison et al., 2019; Islam et al., 2019; Mavhura and Mushure, 2019; Kamwi et al., 2020; Keats and Evans, 2020; Owusu-Ansah, 2020). It has a broader scope of contributing to several of the United Nations Sustainable Development Goals (e.g. SDG 1, 2, 3, 13, and 15) (see Duguma et al., 2018). Specifically, the seeks to answer the following research questions:

1. What strategies do the local people employ in managing the Zukpiri CREMA?
2. What are the benefits of the CREMA to the local people?
3. What challenges do the local communities face in managing the CREMA?

## 2 Empirical and conceptual overview

Africa is among other continents in the developing world that is greatly endowed with natural resources such as water, land and forest landscapes (O'Connor et al., 2021). Africa's development is strongly tied to its ability to effectively manage these natural resources. However, both past and recent efforts to sustainably manage natural resources have not yielded the expected results leading to either overexploitation, mass deforestation and depletion of the natural resources (Husseini et al., 2016; Jarvis et al., 2021). Strategies for managing natural resources are therefore needed to ensure that the natural resource-based of local communities are well managed (Diawuo and Issifu, 2015). Geographic Information Systems and Remote Sensing Technologies, decision support system models, involvement of stakeholders in major decisions regarding natural resource use, community-level initiatives and use of surveillance and monitoring are emphasized as the way forward for effective natural resource management (Amoah and Korle, 2020). Other studies suggested the development of environmental models and strategies that will involve the Payment for Environmental Services (Ansong and Røskaft, 2014). These authors maintained that the surest strategy to adopt in natural resource management is the payment for environmental services (PES). Payment for Environmental Services presents a holistic opportunity for improving natural resource management efficiency and conservation.

Furthermore, collaborative natural resource management strategies emphasizing the integration of local communities in managing natural resources are proposed (Baruah, 2015; Baruah et al., 2016). This notion is embraced with the adoption of community-based natural resource management (CBNRM) approaches as major trajectories to strengthen, empower and motivate local communities to participate in the management of the natural resources found in their localities (Addison et al., 2019). Within the discourse of natural resource management, CBNRM is framed in such a way that it addresses the interest of the local people. It opposes the top-down models earlier used by state institutions in managing natural resources including projects for the development of local communities. As a bottom-up approach to managing natural resources, CBNRM models give greater power and control to the local authorities to decide how and what factors to be put in place in managing their natural environment (Roe et al., 2009). Making local communities part of the management of their natural resources outlines a form of co-ownership and is in tandem with participatory democratic tenets (Roe et al., 2009; Keats and Evans, 2020). According to relevant scholars, CBNRM has been practiced by local communities for several decades in spite of the fact that the mid-1970s marked the period whereby greater attention for it was spearheaded to remedy forest resources degradation and alleviate poverty in developing countries (Wiafe and Arku, 2014).

Another significant CBNRM approach for sustainable management of natural resources involves the adoption of integrated landscape approaches in line with the existing CREMA approach in local communities (O'Connor et al., 2021). The integration of indigenous communities in managing natural resources has attained enviable attention and occurs in diverse forms across different geographies. Jarvis et al. (2021) observed from the operationalization of indigenous land and sea management programmes (ILMPs) that the involvement of indigenous societies in co-managing their natural capital does not only aid in local community development but facilitate and create a platform for the exchange of western and indigenous-created knowledge with a consequential positive impact on environmental quality and societal development. In Nharira Community–Chikomba District of Zimbabwe, Mavhura and Mushure (2019) found that local communities draw cultural and spiritual benefits from natural resources particularly forest reserves and this forms a key element for an informed decision on natural resource management. This explains that indigenous societies have developed approaches for managing their natural wealth based on the purpose and benefits they draw from them. Local norms and customs greatly define the day-to-day forms of natural resources exploitation by the local people (Roe et al., 2009). Also, the reliance on natural resources must be accompanied by defined governance and managerial roles for the local people and the governmental and non-governmental organizations (Roe et al., 2009; Gilli et al., 2020; Jarvis et al., 2021).

In the absence of a defined framework for integrating local people in natural resource governance and/or management, the contribution of these resources to improving wellbeing is mostly threatened (Islam et al., 2019; Kamwi et al., 2020). For instance, in the Greater Serengeti-Mara Ecosystem landscape of Tanzania and Kenya, Jiao et al. (2019) noted that the economic benefits of environmental resources have not been vigorously projected alongside conservation approaches, culminating in various degrees of degradation. This study found a negative association between natural resource dependency and wellbeing. Thus, households that are closer to protected areas highly relied on the resources, cause greater destruction to the physical environment and end up compromising their wellbeing. However, a major strategy to reducing ecological resource dependence by local communities is through protected areas co-management (Islam et al., 2019).

**Figure 1:**
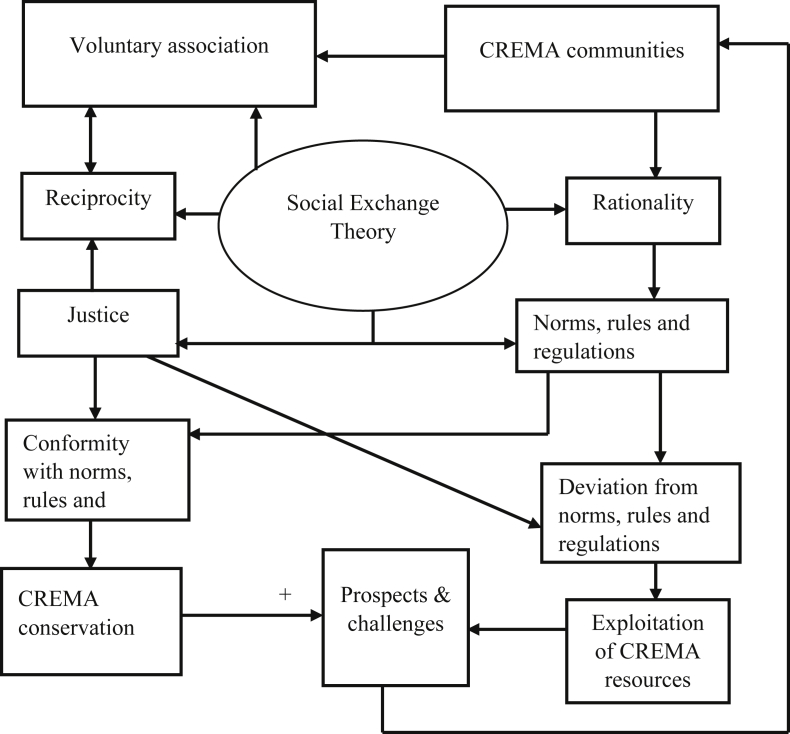
Conceptual framework. Source: Adapted from Bandoh (2010).

Knowledge about the ecological conditions of protected areas such as CREMAs relates to a nearby household decision to conserve the resources. Though nearby households are mostly skeptical about the effects of the future expansion of protected areas, a major factor influencing local communities’ involvement in co-managing the natural resources largely depends on the tangible benefits they derive from these protected areas (Gebregziabher and Soltani, 2019). Hence, maximizing the tangible benefits of protected areas such as CREMAs through the involvement of local communities in co-managing their natural resources must be prioritized. To project the prospects of managing natural resources by local communities, this study draws on the propositions of reciprocity, justice, rationality, norms, rules, regulations and voluntary association as espoused by the social exchange theory to explore the social behaviour of the local communities towards the management of the Zukpiri CREMA. Significantly, attention is given to how community-level norms, rules, and regulations are applied in managing the CREMA (See Figure 1).

Social exchange theory is a social, psychological and sociological perspective that explains social exchange and stability as a process of negotiated exchanges between parties (Homans, 1958). The theory holds the view that human relationships are formed by the use of subjective cost-benefit analysis and the comparison of alternatives. Major proponents of the theory include John Thibaut, Peter Blau, Harold Kelley, Richard Emerson and George Homans. Homans (1958) defined social exchange as the exchange of activity, tangible or intangible and more or less rewarding or costly between at least two parties. Similarly, Blau (1964), explained social exchange as the voluntary actions of individuals that are motivated by the returns they are expected to bring and typically do bring from others.

Social exchange theory emphasized shared responsibilities as showcased in the principles of reciprocity, justice, rationality norms and conformity (Blau, 1964). Thus, co-management of resources between central governments and local communities is pertinent. In addition, sharing of rights and responsibilities through diverse institutional arrangements is encouraged (Roe et al., 2009). In the management of natural resources particularly at the local community level, norms are very vital because they make sure that individuals obey or adhere to the rules and regulations governing the resource use (Diawuo and Issifu, 2015), and in the context of the social exchange theory, conformity to the rules and regulations may lead to a reward while deviation could bring sanctions or punishment to the deviant. In line with the Community Resource Management Area, governed by rules and regulations, any community member found to violate the established rules and regulations regarding the proper resource use and management could be punished according to laid down standards. It is argued that failure to punish offenders will serve as a bad precedent for other community members to emulate which will affect the governance and sustainability of the CREMA (Bandoh, 2010; Owusu-Ansah, 2020). Also, members who abide by the rules and regulations of the CREMA by not illegally exploiting the natural resources in and around the resource area, and avoid bush burning received tangible benefits and the ecological gains of better wellbeing (Bandoh, 2010). Besides, effective management of the CREMA inures to increase non-timber forest products (NTFPs) for the local communities. This study on the prospects of the Zukpiri CREMA will add more insight to a plethora of approaches that can be applied in co-managing natural resources in local communities. Table 2 presents an empirical overview of recent literature on the management, benefits and challenges of natural resource management approaches in related geographies.

**Table 2:** Management strategies, benefits and challenges of natural resource management.

Author(s)/year/title	Research Gap	Methods	Key findings
1. Jiao et al. (2019). Protected areas, household environmental incomes and well-being in the Greater Serengeti-Mara Ecosystem	-Limited knowledge of the importance of environmental resources in protected areas to local households incomes and well-being	-Quantitative methods involving confirmatory factor analysis and multivariate regression analysis were used	-The poor greatly depend on the environment for income. -There is an inverse relationship between reliance on the environment and wellbeing.
2. Mavhura and Mushure (2019). Forest and wildlife resource-conservation efforts based on indigenous knowledge: The case of Nharira community in Chikomba District, Zimbabwe	-There is a dearth of literature on the application of indigenous knowledge in managing and conserving natural resources in protected areas	-Qualitative methods involving focus group discussions and interviews were employed in sourcing data from the local people	-Customary rules and regulations, customs and rituals including totems and taboos were involved in managing, regulating and conserving forest and wildlife resources by the indigenous people.
3. Gebregziabher and Soltani (2019). Exclosures in people's minds: perceptions and attitudes in the Tigray region, Ethiopia	-Enough literature has not been produced on the perception and attitude of people living close to protected areas (exclosures)	-Quantitative methods involving factor analysis, binary logistic regression and multiple linear regression were used to analyse the data	-Socio-economic and ecological factors influence household heads perception and attitude towards exclosures -Household heads have both positive and negative perceptions towards exclosures -There is a strong correlation between household heads' perception and tangible benefits as well as the cost associated with exclosures.
4. Baker et al. (2018). Governance and the making and breaking of social-ecological traps	-The study highlights the connection between formal institutions and informal (customary) institutions in forestalling the continuous natural resource dependence and degradation by local communities	Qualitative methods involving focus group discussions interviews and content/document analysis were applied in the study	-Major causes of resource destruction include overexploitation, endless resource dependence and unsustainable land-use practices -Challenges facing CBNRM paradigms included the low capacity to implement and enforce state policies, natural resource tenure and use conflicts between individuals, communities, and institutions.

Source: Authors.

### 2.1 Evolution of the Zukpiri CREMA and organisational framework

The Zukpiri CREMA was initiated by the Zintang Healers Association, a local traditional healers association, principally to conserve and improve their traditional medicine sources (Zintang Healers Association, 2009). And within a space of time, the richness of the flora and fauna within the CREMA landscape became a major source of attraction to the surrounding farming communities such as Meguo, Siiru, Namuo, Zukpiri, Saan, Nanville, Mantari, Puni, Gudori, Takpo, Kolpeni, Yali, Sampina, Kuuri and Baasi, who willingly organised themselves with the core mandate of sustainably managing the CREMA. As a result, the CREMA received its certificate of devolution on August 19, 2011 (Agyare, 2013). The Zukpiri CREMA remains the main source of medicinal plants for its initiators–the Zintang Healers Association under the monitoring and supervision of the Community Resource Management Committees (CRMCs). The decision to effectively co-manage the CREMA inure to the aforementioned successes attained by the Zukpiri CREMA, the leading CREMA among 18 CREMAs in Northern Ghana (Baker et al., 2018).

**Figure 2:**
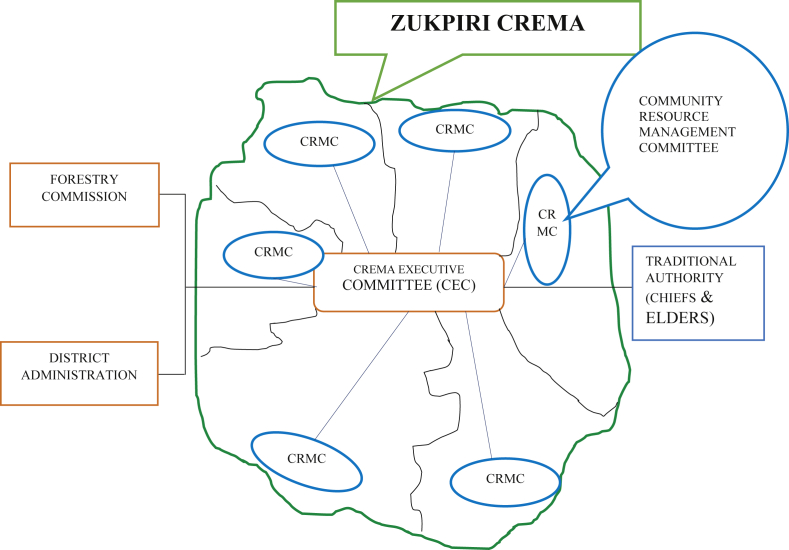
Organizational framework of Zukpiri CREMA. Source: Adapted from Adupong and Gormey (2013).

Community Resource Management Areas (CREMAs) like other CBNRM models operate at various levels in Ghana of which the Zukpiri CREMA is no exception (Asare et al., 2013; Baker et al., 2018). It starts with the CREMA Executive Committee (CEC)–the executive arm and the functional organ of the CREMA (Figure 2). This consists of appointed members from the Community Resource Management Committees (CRMCs) and operates according to the constitution of the CREMA. The next functional unit of the CREMA is the Community Resource Management Committees (CRMCs)–the local unit of the CREMA and exists in every community within the CREMA zone (Agyare 2013; Baker et al., 2018). The CRMCs are formed in accordance with the exiting community administrative protocols, outlining and defining their roles and responsibilities based on the CREMA constitution (Agyare 2013). Furthermore, the coalition of farmers or landholders forms the foundation of the CREMA. They are best described as the shareholders of the CREMA and work hand-in-hand with the CRMCs to execute the CREMA goals (Owusu-Ansah 2020). They also ensure that the executive arm is responsible by using the existing local administrative systems. They are tasked with the responsibility of making as well as amending the constitution to ensure the proper running of the CREMA (Baker et al., 2018).

A CREMA may occur within or beyond one unit committee level depending on the number and spatial distribution of its constituent communities. A Unit Committee is composed of an Assembly member of the electoral areas within which the communities are located and at most five elected members of the area (Asare et al., 2013).

It is however structured that each CREMA is under the care of a CREMA Executive Committee (CEC) but then also has a Community Resource Management Committee (CRMC) within the individual community levels. The CRMCs are formed through community meetings assisted by the Wildlife division of the Forestry Commission. Each community (in other cases two neighbouring communities) may come together to form a Community Resource Management Committee (CRMC) (Asare et al., 2013). During the meetings, the objectives and functions of the CRMC are clearly explained and spelt out to the community members after which nominations for membership are called for through a public voting process. During the voting, those community members selected then become the CRMC for that community. The CRMC is tasked with the responsibility of assisting to accomplish the set targets as well as objectives of the CREMA, to implement activities, and to act as the main link between the CREMA Executive Committee (CEC) and the various communities (Asare et al., 2013; Baker et al., 2018).

Furthermore, each CRMC nominates one person to serve on the CREMA Executive Committee (CEC). It is however designed that the core and voting membership of the CEC is made up of only the local people. However, some provisions are made for representatives of the Wildlife Division of the Forestry Commission, the District Assembly, and any other relevant agencies and organisations that the CEC may deem necessary to be contacted for advice when the need arises (Baker et al., 2018). On the other hand, membership of the CREMA Executive Committee can vary from one CREMA to the other. Governance is assisted by local policies and institutions which include the CREMA constitution framed by communities, led by CECs and shaped by the bye-laws of the District Assembly (Asare et al., 2013; Baker et al., 2018). The Wildlife Division within the Forestry Commission is the primary agent for CREMA development in Ghana. Being a conservation-focused institution with limited rural development power, the Wildlife Division only provides for the conservation site of the CREMAs. Furthermore, the Forestry Commission, the District Assemblies and other relevant Civil Society Organisations equally contribute their quota to the process of CREMA development and the continuous conservation of the diversity of flora and fauna (Agyare 2013; Asare et al., 2013; Baker et al., 2018).

## 3 Materials and methods

### 3.1 Description of the study area

**Figure 3:**
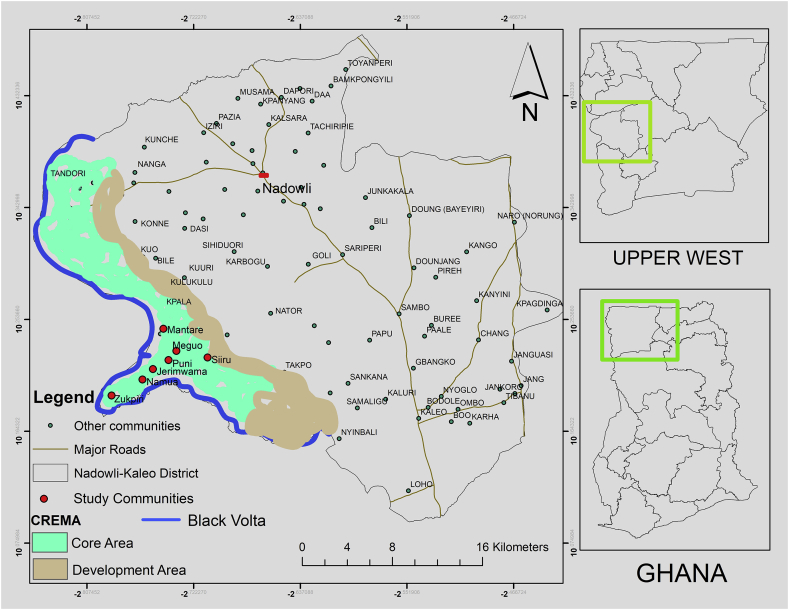
Map of Nadowli-Kaleo Districtleft. Source: Authors construct (2018).

The Zukpiri CREMA is one of the certified CREMAs sited in the Upper West Region of Ghana and is the focus of this study taking into consideration its rich diversity of flora and fauna. In addition, the CREMA also extends into the Black Volta River giving it an exceptional feature of combining forest, wildlife and aquatic resources on the same landscape (Baker et al., 2018). The CREMA is located in the Nadowli-Kaleo District of the Upper West Region (Figure 3). It covers an area of about 420km^2^ and extends from latitude 10.00° to10.20° north and longitude 2.30° to 2.50° west (Ghana Statistical Service, 2014). The CREMA is situated 30km south of Nadowli, the District capital and east of the Black Volta River which forms the Ghana-Burkina Faso international border. About 16 communities surround the CREMA: Takpo, Meguo, Siiru, Zukpiri, Nanville, Mantari, Gudori, Kuuri, Saan, Kolpeni, Puni, Namuo, Yali, Sampina and Baasi. Zintang Healers Association, a traditional healers association promulgated the establishment of the CREMA (Zintang Healers Association, 2009). The CREMA is currently managed by CREMA Executive Committee comprising of representatives of the CRMCS and chaired by a local chief (Asare et al., 2013).

The Nadowli-Kaleo District holds a typical rural economy dominated by the agricultural sector (85 percent). Thus, the inhabitants of the CREMA communities are peasant farmers who focus on the cultivation of food crops like maize, millet, groundnuts, soya beans, sorghum and rice (Ghana Statistical Service, 2014). However, with the inception of the CREMA, some of the farmers have resorted to incorporating tree crops such as mango and cashew on their smallholder farms (Asare et al., 2013). Besides, the inhabitants domesticate livestock like goats, sheep, pigs, and fowls. Additionally, the local craft industry has been vibrant. The men engage in blacksmithing, smock/cloth weaving, and wood carving, while the women engage in basketry, pito brewing, pottery, and shea butter extraction (Ghana Statistical Service, 2014).

### 3.2 Data collection

The study employed a cross-sectional survey design to examine the management strategies, challenges and benefits of the Zukpiri Community Resource Management Area. Data for the study were collected within three (3) months period (August–October 2018), using quantitative and qualitative methods. Multiple sampling techniques were deployed in selecting the study respondents. The first stage of sampling involves a selection of seven (7) communities out of the sixteen (16) CREMA communities. The selected communities include Siiru, Meguo, Puni, Mantare, Jerimwama, Namuo and Zukpiri (Figure 3). These communities were purposively selected because of their closeness to the CREMA.

A sample size of 190 household heads at a 95 percent confidence level was computed using Yamane's (1967:688) sample size determination formula. The sampling frame of 364 household heads (Table 3) was obtained using the Ghana Statistical Service (2014) household lists of the Nadowli-Kaleo District. The obtained sample (190) was distributed proportionally across the seven (7) communities (Table 3). Furthermore, the study employed the simple random sampling technique by using the households' lists of each community and assisted by an excel tool pack, random numbers were generated for the households. Household heads were interviewed because of their high influence on decision-making at the household level and beyond (Baddianaah et al., 2020; Osumanu, 2020). Thus, the household heads' responses regarding the management, challenges and benefits of the Zukpiri CREMA were solicited using a semi-structured questionnaire divided into three major sections. The first section of the questionnaire detailed the respondents' socio-demographic variables, the second section contained issues relating to the management and challenges of the CREMA and the third section contained issues relating to the benefits of the CREMA. The questionnaire administration for the household heads was done devoid of the sex of the household head. Hence, both male and female household heads were involved in the survey. Moreover, in the event of encountering multiple households within a housing unit, the lottery method was used in selecting the appropriate household head for the survey.

**Table 3:** Sample distribution of respondents by communities.

Name of community	Number of Households	Sample size
Siiru	118	62
Meguo	40	21
Puni	38	20
Mantare	49	26
Jerimwama	36	18
Namuo	34	17
Zukpiri	49	26
**Total (Sample frame)**	**364**	**190**

Source: Authors based on Ghana Statistical Service (2014).

Furthermore, the qualitative data were sourced through focus group discussions (FGDs), key informant interviews (KIIs) and field observation. Seven (7) focus groups consisting of seven (7) to eight (8) participants (gender inclusive) were conducted -one (1) in each sampled community). Both males and females constituted the focus groups because the researchers depended on the existing community resource management committees in the selected communities. However, considering the fact that women find it difficult to express themselves in the mix of their husbands, the interviewers ensured that the challenge was addressed by directing relevant questions to women who participated in the survey. A similar composition of focus groups has been constituted by Murray and Agyare (2018) in a related study. The main subject of the discussion was tailored towards the CREMA management strategies and challenges, and the benefits community members derived from the CREMA. The focus groups were conducted in *dagaare* (the native language) and audio recorded after verbal consent was obtained from the respondents. Subsequently, the recordings were translated by the researchers into English Language. All the researchers were fluent in *dagaare,* and this helps in probing further for the local people's perceptions regarding the benefits of the CREMA. Furthermore, the chairperson of the CREMA Executive Committee (CEC)–a chief was interviewed in connection with the management, challenges and benefits of the CREMA. The interview lasted for about 30 min. The researchers also observed and took photographs in the study communities on the CREMA management strategies and benefits. Moreover, respondents' consent was sought whereby an educated community member (Assemblymember) volunteered to read and interpret the research purpose to participants who could not read nor write before the consent forms were endorsed. Furthermore, the household survey questionnaire was vetted and approved by the Ethics Committee of the University for Development Studies. In addition, though the respondents were assured of confidentiality, their withdrawal from the survey at any point in time was guaranteed.

### 3.3 Data analysis

The household (quantitative) data were coded and entered into Statistical Package for Social Sciences (SPSS version 20.0) spreadsheet and simple percentages were generated for relevant variables. The results were presented in tables and figures. The qualitative data were analysed through thematic and content analysis). Responses from the focus groups and key informants were recorded and transcribed according to the dominant themes (Charmaz and Belgrave, 2012). Furthermore, the transcripts were screen and organised according to the salient themes for presentation. This helps eliminate all irrelevant discussions and ensures that the research aim is specifically addressed. Finally, the data were presented using texts comprising direct and indirect quotes.

## 4 Results

### 4.1 Socio-demographic characteristics of respondents

The results (Table 4) showed that the majority (61.1 percent) of the respondents were males of which 52.6 percent of them were within the age group of 30–39 years, and the aged (60+) were the least represented (1.1 percent). The majority (89.5 percent) of the respondents had no formal education with a small proportion (3.2 percent) of them having completed Junior High School. Also, the occupational status of the respondents showed that a majority (99.5 percent) engaged in agriculture and its related activities. Almost all respondents (98.9 percent) were indigenous settlers.

**Table 4:** Sex distribution of respondents (N = 190).

	Frequency	Percent
**Gender**		
Male	116	61.1
Female	74	38.9
**Age group**		
20–29	32	16.8
30–39	100	52.6
40–49	43	22.7
50–59	13	6.8
60+	2	1.1
**Educational status**		
No formal education	170	89.5%
Primary school	14	7.4
Junior High School	6	3.2
**Occupation**		
Farmer	189	99.5
Trader	1	.5
**Membership**		
Indigene	188	98.9
Non-indigene	2	1.1

Source: Field Survey (2018).

### 4.2 Strategies employed in managing the Zukpiri CREMA

A leading step towards managing the CREMA is the formation of the community resource management committees (CRMCs). The presence of these committees was acknowledged across all the study communities. These committees exercise several responsibilities in line with their duties. The committee members are assigned duties and responsibilities of which people are nominated on weekly basis to constantly patrol, monitor, track and arrest people who flout the rules and regulations and engage in illegal harvesting of the CREMA resources. Respondents indicated that they exercise their duties voluntarily by patrolling the CREMA core zone day and night. This is done without any salary or immediate reward. However, in the event of any financial support from the government, governmental organisations or Non-Governmental Organisations (NGOs), active members stand to benefit the more.

The CREMA committees are made of seven (7) members in each community. However, the chief (*Naa*) and landlord (*Tindana*) are automatic members of the CRMC where the chief becomes the chairperson in every community. The other five (5) members within each community are selected based on consensus mostly through a durbar convene by the chiefs and elders. Notably, a significant gap in terms of the CREMA committees’ composition at the local level had to do with the fact that members have no definite tenure of office – “till death do us apart”. A discussant intimated:

*We don't change the membership of the CREMA committees like that, unless there is gross misconduct or death of a member, that is, till death do us apart. You see, the CREMA has several implications, land tenure, and even the age of the representatives are all issues we consider in constituting the committees. We need people that are stable, focused and committed to the work. And a majority of the youth cannot meet all these criteria* (Source: Key Informant Interview with a Chief, July 2018).

The next stage of the CREMA management involves the composition of the CREMA Executive Committee (CEC). In constituting the CREMA Executive Committee (CEC), each community nominates a representative from the host Community Resource Management Committee (CRMC). The CREMA Executive Committee (CEC) meets on monthly basis to deliberate on key strategies to improve and sustain the CREMA. Management of the CREMA involves setting up well defined rules and regulations such as *“no hunting, no cutting of trees, and no burning of charcoal, no fishing, no bush fire and no grazing in and around the CREMA”.* These are the fundamental principles that guide the effective management of the Zukpiri CREMA. Besides, the chiefs and elders of the CREMA communities employ norms, beliefs, totems and taboos to protect and conserve the resources found in the CREMA. An elder in a focus group said:

*“Effective management of the CREMA relies on many factors but I will say the aspect of traditional beliefs is perfectly working in the protection of the CREMA resources… animals such as the python, hippopotamus, crocodile, hedgehog, and a whole lot are totems of the various communities and one dare kill the totem of the other”* (Focus group discussion, October 2018).

A member who violates any of the bye-laws is punished accordingly. The nature of the punishment to be given to an offender varies but appears to be punitive enough. A discussant had this to say: *If someone enters the CREMA to hunt and is caught, he/she pays an amount of GHC 500.00 with his gun and other hunting tools confiscated. Also, the person is made to sign a bond to be of good behaviour after paying the fine* (Focus group discussion, August 2018). The communities used their ingenuities and innovations to maintain and prevent the CREMA from wildfires that frequently occur in the dry season. This is achieved by creating a fire belt around the entire CREMA zone. The timing is important in this regard, and as a result, the fire belt is created between October and November every year. According to a discussant:

*“a day is usually fixed by the chiefs and elders of which every member of the community is made aware. This often results in a higher turnout since all able body men and women are mandated to take part in the creation of the fire belt. And failure to participate in creating the fire belt attracts a fine of fifty Ghana Cedis”* (approximately 9 dollars–1 dollar = 6 Ghana Cedis as of August 7, 2021) (Focus group discussion, August 2018).

**Figure 4:**
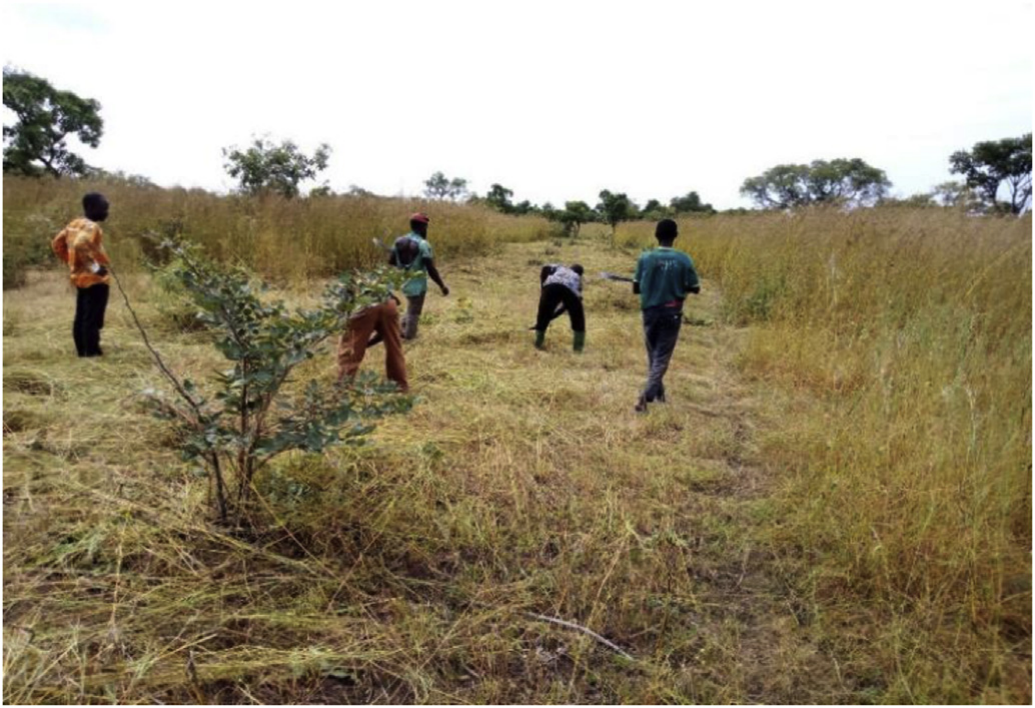
CREMA community members creating a fire belt (Date taken: 15th October 2018). Source: Field Survey (2018).

Our visit coincided with the creation of a fire belt by a section of the CRMC (Figure 4).

### 4.3 Contribution of the Zukpiri CREMA to livelihoods

Although the communities have reservations about staying close to the CREMA, the CREMA has impacted positively on their livelihoods in terms of contribution to household food supplements, income and the general wellbeing of the household. The results revealed that some household heads earned income from the CREMA (Table 5). The CREMA appears to be a source of income generating portfolio for women. A discussant submitted that: *women gather firewood, shea nuts, dawadawa, mushrooms, medicinal plants leave, fruits and other non-timber forest products that go into the meals of their families or are sold in the local markets to generate income* (FGD, 2018). The CREMA communities are reaping the benefits of the CREMA in many ways besides selling the NTFPs in the local markets.

**Table 5:** Income earned from the CREMA.

	Frequency	Percent
**Earn income**		
Yes	56	29.5
No	134	70.5
**Income used**		
No income earned	134	70.5
To buy cooking utensils	4	2.1
Used it to buy foodstuffs	10	5.2
It is used to purchase a tricycle	1	0.5
Payment of school fees and health insurance	26	13.8
Use to buy animals for rearing	15	7.9

Source: Field Survey (2018).

Another discussant intimated:

*It will be unfair for anyone in this village to say the CREMA is not contributing to his or her household's income. Take the women, for instance, they were taken through how to use the moringa leaves to prepare soap by an NGO. Again, we were all trained on how to process and package the moringa leaves into tea bags and those of us that took it seriously are reaping the benefits today. Our main problem in this village is uncertainty which is killing us. We have the resources at our disposal through this CREMA but a majority of us doubt the prospects of making money from it…we only know the destructive aspects, colluding with strangers to illegally fell rosewood as the quickest means of making money from the CREMA* (FGD, August 2018).

Respondents who earned income from the CREMA used their incomes in diverse ways. Some used it to pay their children school fees (13.8 percent), others used it to purchase livestock for rearing (7.9 percent) while others used it to buy foodstuffs (5.2 percent) to feed their families. Notably, one person (0.5 percent) was able to accumulate income from the CREMA and used it to purchase a tricycle to facilitate his means of transport (Table 5).

Respondents indicated that they are reaping the benefits of the CREMA in many folds that they did not anticipate during the inception of the CREMA. Out of the 190 household heads interviewed, 37.9 percent benefitted from the CREMA through an NGO that trained them on value addition to some of the CREMA resources. These pieces of training were on the preparation of soap, preparation and packaging of local tom brown, preparation and packaging of dawadawa tea and moringa tea. The community members (20.5 percent) indicated that the inception of the CREMA has improved the rainfall pattern, thereby, facilitating their agricultural activities. Also, 15.8 percent of the respondents indicated that their community had a school block, a borehole and a grinding mill all through the influence of the CREMA. Further, 13.7 percent of respondents indicated that the CREMA has attracted the attention of governmental organisations (e.g. United States Agency for International Development [USAID], United Nations Development Programme [UNDP]) and many more who supported them with several items like bicycles, motorbikes, mobile phones, solar lights and in some cases cash (money) (Table 6).

**Table 6:** Indirect benefits derived from the CREMA.

Unexpected Benefits	Frequency	Percent
Dangerous animals like snakes have moved into the forest (CREMA)	2	1.0
We got items such as bicycles, motorbikes, mobile phones, solar lights and money	26	13.7
Construction of school block, drilling of bore-hole, reshaping of roads, and a grinding mill.	30	15.8
It induces rainfall	39	20.5
Some equipment for butter and moringa processing.	3	1.6
Some people get employed in the CREMA	1	0.6
Through the CREMA, we have been given some form of education on farming.	13	6.8
The CREMA serves as a tourist centre	4	2.1
Training of our women on how to prepare soap, local tom brown, dawadawa tea and moringa tea	72	37.9

Source: Field Survey (2018).

**Figure 5:**
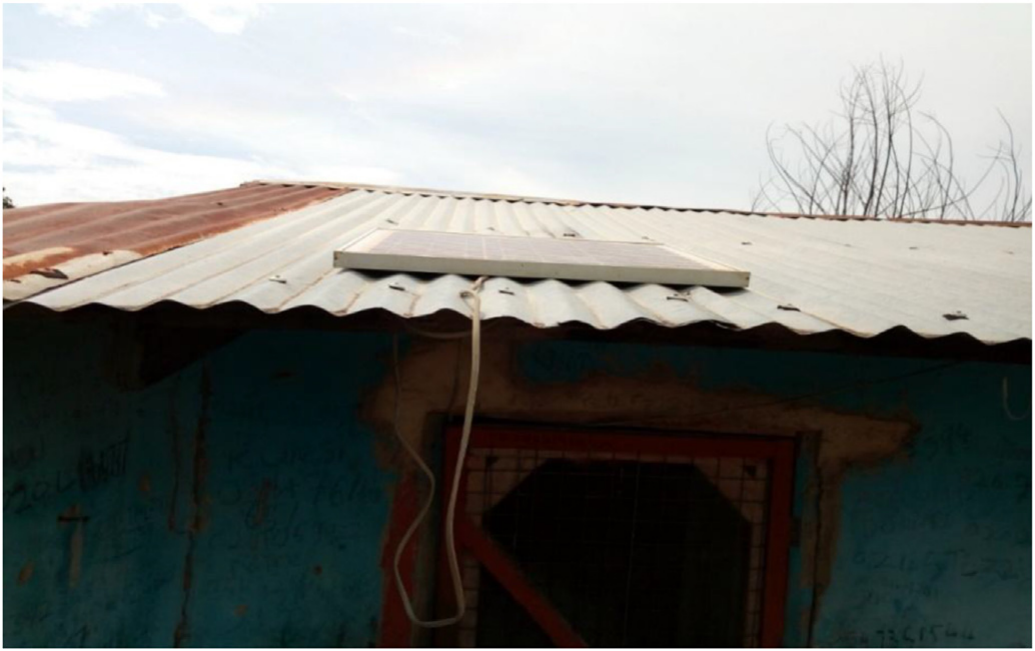
A Solar Panel mounted on the roof of a beneficiary (date taken: 13^th^ October 2018). Source: Field Survey (2018).

This was corroborated by responses from a key informant who opined:

*The CREMA has brought a lot of benefits to us. For example, some community members were selected and send to India for three months and these people were trained on solar installation and repairs, they came home with solar products such as lamps, radio sets and these products are still been enjoyed by us today. The one on my roof is evidence of what I am talking of* (see Figure 5)*. We consider the CREMA as something God planned to bless us with* (Source: Key Informant Interview with a chief, August 2018).

### 4.4 Challenges faced in managing the CREMA

The challenges facing the community members in managing the CREMA are numerous. The Community Resource Management Committee (CRMC) members indicated a lack of protective clothes, torch lights and cutlasses to enable them properly monitor the CREMA. They indicated their members, that is, those entrusted with monitoring and surveillance of the CREMA wore protective clothes to prevent them from insects’ bite and also take cover from the (cold) weather particularly during the harmattan. Significantly, the CREMA is faced with land tenure conflicts and conflicts over the use of the resources found within the CREMA. The discussants indicated the land under which the CREMA is occupying was their farmlands of which they have been deprived of these lands without any form of compensation. Farmer herder conflicts and conflicts with herders and CREMA communities came up in the discussion. That aside, it was discovered that the CREMA zone has been extended beyond its initial limit. This, according to the discussant has brought the CREMA too close to their communities, restraining the dwellers from expanding the compound farms. Besides, the nearness of the CREMA to the local communities sets in conflicts between the community members and the CREMA management committee members. A discussant opined:

*There is a tug of war between us and the community members. One, we prevented them from grazing their animals in the CREMA but if you look at it carefully, we are also infringing on their rights in a way because the communities that are too close to the CREMA have their grazing lands taken away by the CREMA. In the event a committee member kills a domestic animal that enters the CREMA, it usually results in reprisal attacks on the innocent wildlife of the CREMA. I mean they kill the animals in the CREMA as payback* (Focus group discussion, October 2018)*.*

## 5 Discussion

The results showed that household headship in the CREMA communities is dominated by men (Table 3) and reflects a dominant feature of household leadership in local communities in northern Ghana (Baddianaah et al., 2020; Osumanu, 2020). With the household head being the main decision-making agent regarding the entire wellbeing of the household (Osumanu, 2020), males have a greater chance of influencing decision-making regarding the CREMA management and exploitation of the resources. The majority of the study respondents have not attained formal education–the ability to read and write (Ghana Statistical Service, 2014). The low level of formal educational attainment of the respondents (see Table 4) may have implications on their understanding of other benefits of the CREMA aside from the harvesting of non-timber forest products. The decision to conserve the CREMA resources is likely to be influenced by the immediate benefits or perceived benefits they stand to gain from the CREMA. This finding corroborates the finding of Abukari and Mwalyosi (2018) who found that knowledge regarding the rules and regulations of conservational parks (Mole National Park, Ghana and the Tarangire National Park, Tanzania), and related benefits like employment creation, access to non-timber forest products (NTFPs) and livelihood diversification greatly inform local communities conservation decision. The respondents are largely peasant farmers who relied on their local knowledge in protecting and conserving the CREMA resources. Thus, the use of indigenous knowledge in conserving natural resources has proven to be useful across local communities (Asare et al., 2013; Bandoh, 2010; Yahaya, 2012; Husseini et al., 2016; Abukari and Mwalyosi, 2018; Addison et al., 2019; Mavhura and Mushure, 2019; Jarvis et al., 2021).

With guidance from the Forestry Commission of Ghana, the communities formed the Community Resources Management Committees showcasing a collaborative natural resource management trajectory. These committees are recognised across all the communities as the leading decision-making organisations concerning the CREMA. The Committee members demonstrate a high level of commitment in ensuring that the rules and regulations of the CREMA are adhered to by all community members. This explains that the use of local rules and regulations have great impact on natural resource protection and conservation. The creation of bye-laws aimed at protecting and sustaining the CREMA corroborates the conceptual perspective of using norms and conformity in natural resource management. Norms are very vital in every society because they make sure that individuals adhere to rules and regulations of society. Duties and responsibilities are initiated by the CRMCs where community members willingly participate in the monitoring and protection of the CREMA (see Figure 4). One significant measure of sustainably managing natural resources by local communities is the willingness of the inhabitants to adhere to the rules and regulations as well as participate in protecting and conserving the resources (Asare et al., 2013; Bruah, 2014; Owusu-Ansah, 2020). However, they expect a fair distribution of whatever benefit that may arise for the better management of the CREMA. The use of fines and other forms of punishment to deter community members from illegally harvesting the CREMA resources appears to be working out for the Zukpiri CREMA. For instance, the Zukpiri CREMA is well managed and has a lot of accolades to its credit. In 2015, the Zukpiri CREMA was the 2^nd^ runner-up in the United Nations Development Programme (UNDP) Global Environmental Award in Washington D.C., USA. Again in 2016, the United States Agency for International Development (USAID) evaluation of CREMAs in Northern Ghana rated Zukpiri CREMA first out of the 18 CREMAs in the three Northern Regions (Baker et al., 2018). This suggests that strict enforcement of rules and regulations coupled with hard fines are convincing means of protecting illegal exploitation of natural resources in local communities. The logistics and land tenure constraints uncovered in this study appear to be common among local communities that collaborate with state institutions to conserve natural resources (Asare et al., 2013; Baruah, 2015; Baker et al., 2018; Gilli et al., 2020; Owusu-Ansah, 2020). Since the CREMA model emphasised collaboration between the responsible state institutions like the Forestry Commission and the local communities, state institutions must work in line with addressing the challenges facing the communities.

Staying close to the CREMA comes with positive and negative ramifications (Table 6). However, the positive effects of CREMAs are encouraged (Abukari and Mwalyosi, 2018). Women harvest non-timber forest products from the CREMA to support their household food basket. This is in tandem with the burgeoning literature on the contribution of CREMAs and protected areas to livelihoods in local communities (Abukari and Mwalyosi, 2018; Islam et al., 2019; Kamwi et al., 2020; Osei-Wusu et al., 2020). The shea for instance has been heavily depended upon by the majority of the women in the CREMA Communities for income. The fruits are consumed directly, while the nuts are further processed into shea butter. Shea butter has several uses in the cosmetic, and pharmaceutical industries (Gilli et al., 2020). The natural healing power of the shea cannot be downplayed. It heals sores, burns and scars when applied directly (Zintang Healers Association, 2009). This suggests that the shea has been the major livelihood backbone of the CREMA communities in northern Ghana. However, the income sourced from the CREMA is used in many ways including paying school fees, purchasing household cooking utensils and paying for the cost of health care needs of the household (Table 6). Also, several medicinal plants are harvested from the CREMA all of which go a long way to improve the livelihoods of the CREMA communities. In the specific instance of the Zukpiri CREMA, it was initiated by the Zintang Healers Association purposely to support their traditional medicine sources and has since remained a major source of herbs to many people (Zintang Healers Association, 2009; Baker et al., 2018).

Besides the direct benefits the communities draw from the CREMA in the form of harvesting non-timber forest products, they received many gifts and rewards for taking up the task of properly managing the CREMA (see Table 6 and Figure 5). This comes in the form of cash (money) from NGOs and CSOs, entrepreneurial training on adding value to the resources found within the CREMA in line with sustainable exploitation and consumption of natural resources. Additionally, some community members had the opportunity to travel outsides Ghana because of their roles and commitment to managing the CREMA. The CREMA has attracted the attention of relevant governmental organisations such as UNDP, USAID, and UKAID who came in to address some of the basic challenges facing the communities by providing them with school buildings and potable drinking water systems (see Table 6). The CREMA has projected the communities on the global map. These forms of assistance offered to the CREMA communities have not been strongly reported in the growing literature on the contribution of CREMAs to livelihoods in local communities. Community Resource Management Areas (CREMAs) occur on the objective of environmental conservation and are in line with reducing poverty among the local people (Duguma et al., 2018). The CREMA is therefore considered as a developmental tool to the local communities (Gilli et al., 2020). Additionally, wildlife such as the hippopotamus, crocodiles, and monkeys found in the CREMA received constant visitation by both local and international tourists. This corroborates the findings of related studies citing CREMAs and protected areas as significant tourist sites (Bandoh, 2010; Oduro et al., 2012; Osei-mainoo, 2012; Abukari and Mwalyosi, 2018; Mavhura and Mushure, 2019; Jarvis et al., 2021). In tandem with the aforementioned prospects of the Zukpiri CREMA to the local communities, the study, therefore, argued that besides creating an enabling ecological environment (Abukari and Mwalyosi, 2018; Duguma et al., 2018; Mavhura and Mushure, 2019; Baskent, 2020), inhabitants of CREMA communities are reaping the benefits of the CREMAs in many folds. Hence, the more communities enjoy these benefits from the CREMAs, the more they will commit their attention to protecting, managing, and conserving the natural resources found within their sphere of influence. However, issues of natural resource use conflict among community members and conflict with Fulani herders over the CREMA resources need further interrogation.

## 6 Conclusion

The study examined the management strategies, benefits and challenges of the Zukpiri CREMA in the Upper West Region. Inhabitants of the Zukpiri CREMA employ diverse plans and management strategies in protecting, managing and conserving the natural resources found within the CREMA. They among others rely on local knowledge systems to effectively manage the CREMA. Despite the fact that the communities are constraint in terms of logistics to effectively perform their duties of monitoring and supervision for effective management of the CREMA, it has contributed tremendously in diverse ways to the livelihood of the dwellers and the entire developmental status of the communities. The CREMA is a source of food and income for many households. Women collect non-timber forest products (NFTPs) predominantly the shea which has several uses and prospects to the communities. Furthermore, the CREMA provides medicinal herbs to the local people including the initiators of the CREMA–Zintang Healers Association whose traditional medicinal needs brought the CREMA into existence. Again, the CREMA attracts tourists, and also placed the communities on the global map for winning the best managed CREMA among the 18 CREMAs located in Northern Ghana. In connection to this achievement, the CREMA has attracted the attention of NGOs and CSOs to the communities with their support coming in the area of providing school buildings and potable water systems, offering local communities entrepreneurial training, cash distribution to committed members of the CRMC including taking some community members abroad for further training on solar light installation. In line with the aforementioned prospects of the CREMA to the local communities, this study argued that besides creating an enabling ecological environment, inhabitants of the CREMA communities are reaping the benefits of the CREMA in many folds. Hence, the more communities enjoy these benefits from the CREMA, the more they will commit their attention to protecting, managing, and conserving the natural resources found within their sphere of influence. The study, therefore, implores the Forestry Commission of Ghana to focus on exploring more livelihood alignment programmes in tandem with the CREMA approach.

## Declarations

### Author contribution statement

Louis Baaweh & Issah Baddianaah: Conceived and designed the experiments; Performed the experiments; Analyzed and interpreted the data; Contributed reagents, materials, analysis tools or data; Wrote the paper.

### Funding statement

This research did not receive any specific grant from funding agencies in the public, commercial, or not-for-profit sectors.

### Data availability statement

Data will be made available on request.

### Declaration of interests statement

The authors declare no conflict of interest.

### Additional information

No additional information is available for this paper.
